# Ecomorphological Variation of the Wireworm Cephalic Capsule: Studying the Interaction of Environment and Geometric Shape

**DOI:** 10.1371/journal.pone.0102059

**Published:** 2014-07-08

**Authors:** Hugo A. Benítez, Thomas Püschel, Darija Lemic, Maja Čačija, Antonela Kozina, Renata Bažok

**Affiliations:** 1 Faculty of Life Sciences, University of Manchester, Michael Smith Building, Oxford Road, Manchester, United Kingdom; 2 Instituto de Alta Investigación, Universidad de Tarapacá, Casilla, Arica, Chile; 3 University of Zagreb, Faculty of Agriculture, Department for Agricultural Zoology, Svetošimunska, Zagreb, Croatia; Laboratoire Arago, France

## Abstract

Studying the association between organismal morphology and environmental conditions has been very useful to test hypothesis regarding the influence of climate on shape. It has been long recognized that different environments produce dissimilar stress levels in insects, which can be reflected on the ability of an individual to overcome these pressures and spread further. *Agriotes* (Coleoptera: Elateridae) species infest agricultural fields in different parts of Croatia, inhabiting different climatic conditions. Previous biological studies have indicated that there is a relationship between some *Agriotes* biological parameters such as density and climatic conditions such as soil moisture and temperature. However, it is still unknown how these environmental properties influence the wireworm morphological structure. This is highly relevant because the head of this species is directly involved in the mobility in the soil, thus affecting the invasive capacity of this insect. Therefore the aim of this study was to assess the association between different climatic conditions and the morphological variation of *Agriotes* cephalic capsule. Advanced multivariate analysis and geometric morphometric tool were applied to study the covariation between shape and environmental variables. Partial Least Squares methods were used in order to analyse the association between the wireworm head shape and three different climatic conditions: soil type, temperature and rainfall. Our results showed that there is a high covariation between the wireworm head shape and the climatic conditions. It was suggested that the observed shape–environment association could be result of the high plasticity of this species in relation to its invasive capacity.

## Introduction

Taxonomic classification and biological diversity analyses have been traditionally based on morphological descriptions [1]. Driven by a mathematical quantitative revolution, morphological studies have experienced a significant revitalization due to the development of shape analysis based on statistical multivariate techniques and novel visualisation methods. Broadly, morphometrics refer to the quantitative analysis of form (i.e. shape and size) and how it covaries with respect to other factors (e.g. ecology, development, biomechanics, and genetics among others) [2]–[5]. Geometric morphometrics (GMM) is a coordinate-based method, which means that their primary data are 2D or 3D Cartesian coordinates of anatomically distinguishable landmarks (i.e. discrete anatomical loci that are arguably homologous among all the individuals under analysis). Coordinates are better when compared to linear data, because they preserve the spatial information of a structure, providing a relatively complete description of an organism’s shape [2], [3], [6]. One of the great characteristics of GMM is that it allows studying the association between shape and other kinds of data, such as ecological, genetic, biomechanical, or other relevant factors. This is really useful because one of the traditional interests of ecologists is to associate character states or different phenotypic values with environmental data. There are several matters for which morphological analyses play an important role in ecology. For instance, ecomorphological studies have revealed constraints and selective factors affecting the phenotypic response to certain environments [7]–[9], how morphology influences the ecological distribution of a particular phenotype [10]–[13] and evolutionary trends such as phylogenetically conserved morphologies [11], [14]–[16]. In all these cases, morphology represents certain organismal aspects that relate and individual to its environment, hence its importance. Indeed, the association between morphology and ecology could provide useful insights about the expression of the phenotype-environment interaction and the related evolutionary history. There is a plethora of available methods to study association between morphological and ecological variables (e.g. regression analysis; canonical correlation analysis; Mantel test; principal coordinate analysis; etc.). However, Partial Least Squares (PLS) method is probably one of the mostly applied when assessing the covariation between shape and other factors [17]. For example, it has been used to relate morphometric and ecological variables [5], [13], [18]–[20], morphometric and allele frequency data [21], shape and behavior [18], different parts of the same configuration of landmarks [21]–[23], and even data from different parts or different views of the same specimens [24], [25].

Wireworms are click beetle larvae from the genus *Agriotes* (Coleoptera: Elateridae) that considerably damage field crops, especially potatoes [26]–[28]. They are long-lived soil insects; most species spend 3–5 years in the larval stage [26], [28]. Their body are elongated and hard, characterized by a reddish-brown coloration [29]. They are polyphagous and usually inhabit most kinds of soil. Some wireworm species are serious agricultural pests [28], [30], [31]. Larvae feed on germinating seeds and plant roots, affecting negatively seedlings and young plants, often causing their death [32], [33]. Among over 10,000 species known worldwide [29], there are just around 150 considered dangerous enough to cause significant damages to agricultural cultigens. The five most important *Agriotes* species in Croatia include *A. lineatus* L., *A. sputator* L., *A. obscurus* L., *A. brevis* Cand. and *A. ustulatus* Schall. They can produce significant economic losses in agriculture, due to their feeding behaviour [34]. *Agriotes* “species infest agricultural fields and cause serious economic damages in the continental part of Croatia. Therefore, studying these species is relevant due to the impact that they have on agricultural production.

East continental Croatia is characterized by chernozemic soils, while the central region is defined by ground water gley and alluvial muds soils, and the west part is characterized by luvic and pseudoglay soils [35]. As a result of the dissimilar soils found in each one of these regions, the prevailing microhabitat parameters are different. The most important parameters are soil moisture and temperature, both of which are known to impact upon oviposition, egg survival and larval development [27], [30], [34], [36]. Unlike the other four species, *Agriotes ustulatus* overwinters only as larvae and develops for two (three calendar) years [27], and the higher temperatures positively affect its abundance [27], [37]. According to previous literature, *A. ustulatus* was the most frequent species only in the eastern regions of Croatia [32], [38], [39] but recent studies have shown that this species has also spread to other regions of continental Croatia [37], [40].

It is well known that the adaptation over time to a specific environment is the result of both environmental pressures and geographic distance [41]–[43]. Moreover, it is well documented that adverse temperatures, nutritional stresses, presence of chemicals, population density and many other factors that affect development can lead to an increase in morphological asymmetry as a result of high intraspecific variation (e.g. [42], [44], [45]). Therefore, it is expected that when environmental conditions change, organisms and populations should adapt to the new conditions [45]. In this context, adaptive variation plays a key role since it reflects historical evolution and determines the population’s phenotypic response [46]–[48]. Moreover, Bouyer et al. [49] have suggested that the influence of environment on an organism’s genotype takes more time to manifest than on the phenotype, and as such the study of environmental influence on populations and individuals should be made on phenotypic, rather than genotypic, characters. Hence, we preferred the application of geometric morphometric methods instead of molecular analyses, because our goal was to analyse the association between environmental factors and morphological structure, rather than determining the genetic response to different climatic conditions. Previous studies have analyzed the effect of different ecological factors on insect morphology from diverse perspectives, for instance anti-predator defences [50]–[52], behaviour and sexual dimorphism [53]–[57], physiology [58]–[60] and environmental adaptations [61]–[64]. However our approach is innovative, because it is a novel attempt to characterize the relationship between a larval morphology with closely related environmental conditions. The aim of this study was to assess the association between different climatic conditions (i.e. temperature, rainfall and soil type) on the morphological differentiation of *Agriotes ustulatus* cephalic capsule. Furthermore, this study applied for the first time geometric morphometric techniques to analyze beetle larvae shape variation.

## Materials and Methods

### Ethics Statement

“N/A”.

Our samples not needed any specific permission to collect (locations/activities) and this article is not involved in any endangered or protected species.

### Data collection

**Figure 1:**
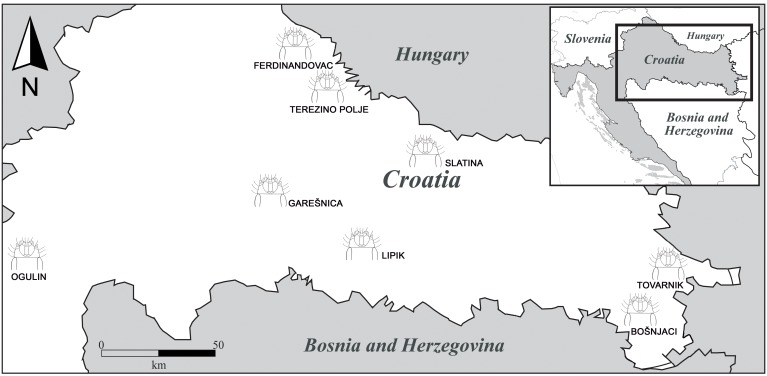
Geographic distribution of the eight Croatian localities of *A. ustulatus* examined in this study.

In this study we analysed morphological differences in 258 wireworms of *Agriotes ustulatus*. The wireworm larvae were sampled during spring (May) and autumn (September) from 2009 until 2013. Although the taxonomic identification of the *Agriotes* species at the larval stage is extremely difficult, the analyzed larvae were positively classified as member of *A. ustulatus* based on the distinctive presence of specific marks on the last abdominal segment. This particular character is an exclusive trait of this species and none of the other members of this genus shows it [65]. They came from eight different locations of continental Croatia. Locations were grouped according to climatic conditions and soil type. Group 1 corresponds to the eastern locations of Tovarnik and Bošnjaci, which had drier weather conditions and chernozemic soils. Group 2 includes Slatina, Terezino Polje and Ferdinandovac, which had lower temperatures as compared to the eastern location but higher moisture and alluvial soils. Group 3 are Garešnica and Lipik locations, which had climate conditions in between the two previous groups and ground water gley soils. Group 4 consisted of one location on the west, Ogulin, which is on higher altitude and had cooler climate (perhumid climate), with luvic and pseudoglay soils (Fig. 1, Table 1). All sampled areas were crop fields, cultivated with oilseed rape, maize, barley, soybean, sugar beet, tobacco, alfalfa and potato. The sampling method consisted in digging a series of ca. five holes (dimensions 25×25×25 cm) on each field [38]. All the excavated soil was manually crumbled to small pieces on a black foil and examined thoroughly to detect any wireworm presence. The collected specimens were preserved in 70% ethanol. Species determination was done by examining the ninth abdominal segment [65]. Only larvae of the approximately same developmental stage (7th–13th instar, larger than 10 mm) of *Agriotes ustulatus* species were selected for further analysis [27]. All the specimens are housed in the Department for Agricultural Zoology, University of Zagreb.

**Table 1:** Localities with their corresponding geographic coordinates and environmental variables: n, number of specimens; T, mean annual between 2009–2013 temperature (°C); Rain, Rainfall (mm); Type of soil (group 1: chernozemic soil, group 2: alluvial soil, group 3: ground water gley soil and group 4: luvic and pseudoglay soil).

Location	Samples (n)	Coordinates	Temperature(Average 2009–2013)	Rainfall(Average 2009–2013)	Type of Soil
a. GAREŠNICA	40	45°34′15.39″N;16°55′56.02″E	11.87	58.83	ground water gley soil
b. LIPIK	35	45°24′59.61″N;17°9′24.10″E	11.90	70.6	ground water gley soil
c. TOVARNIK	37	45°9′24.43″N;19°9′14.51″E	12.35	40.99	chernozemic soil
d. BOŠNJACI	39	45°2′54.64″N;18°44′59.59″E	12.49	60.97	chernozemic soil
e. SLATINA	40	45°42′51.70″N;17°41′59.18″E	11.26	61.57	alluvial soil
f. TEREZINO POLJE	2	45°56′5.47″N;17°27′27.82″E	11.513	71.69	alluvial soil
g. FERDINANDOVAC	13	46°3′42.85″N;17°10′59.92″E	11.078	66.71	alluvial soil
h. OGULIN	46	45°15′27.46″N;15°12′38.78″E	10.96	129.171	luvic and pseudoglay soil

### Landmark Acquisition

**Figure 2:**
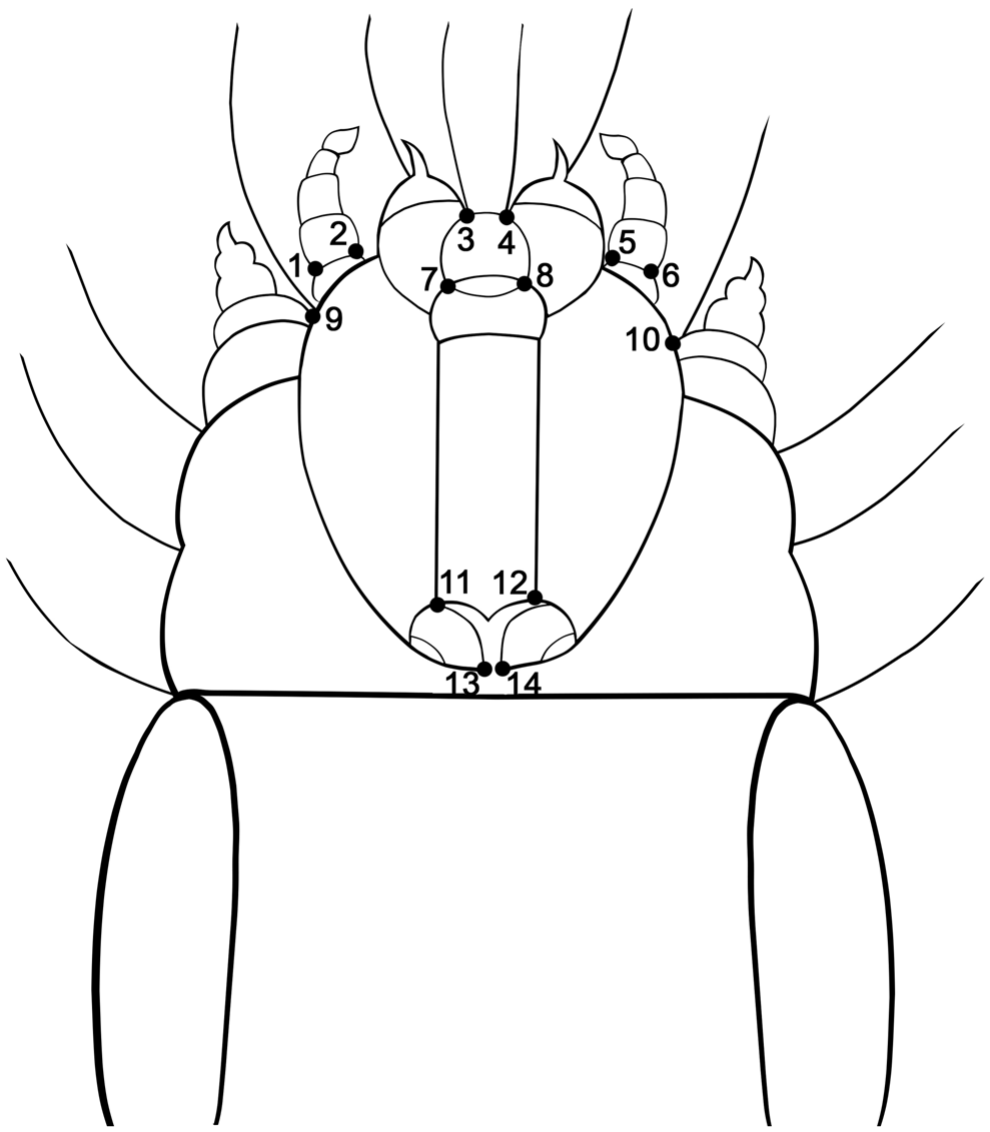
Representation of the 14 morphological landmarks identified on the cephalic capsule of *A. ustulatus.*

Cephalic capsules were photographed using a Leica DFC295 digital camera (3M Pixel) on a trinocular mount of a Leica MZ16a stereo-microscope and saved in JPEG format using the Leica Application Suite v3.8.0 (Leica Microsystems Limited, Switzerland). Fourteen homologue type 1 landmarks (Fig. 2), (Table S1, Table S2) were marked on the wireworm mandible, so that they could be accurately and repeatedly identified. Each landmark was digitised using tpsDIG V2.17 [66] and imported into MorphoJ v1.04d for further statistical analyses [67]. The dataset containing the raw coordinates used in this study is available as supplementary material. Measurement error (ME) has a critical importance when analyzing shape. In order to assess the ME level, the cephalic capsules of 35 individual beetles were digitized twice [68].

### Shape Analysis

Once obtained the Cartesian coordinates for all landmarks, the shape information was extracted by performing a full Procrustes fit [69], [70] taking into account the object symmetry of this anatomical structure. Procrustes superimposition is a mathematical procedure that removes the variation due to rotation, position, orientation and size [69]. Due to the existence of object symmetry in the analysed structure, reflection was removed by including the original and mirror image of all configurations in the analysis and simultaneously superimposing all of them [71].

Allometry is a key factor influencing shape variation [72], [73], therefore allometric effects were assessed by performing a multivariate regression of shape on centroid size, pooling the dataset by location. Then the covariance matrix of the residuals pooled by location was computed to perform the following analyses.

### Multivariate Analysis

The main patterns of variation in the shape space were visualized by carrying out a principal component analysis (PCA), calculated from the covariance matrix of the averaged population symmetric component of shape [71]. In order to statistically test if there were differences between the locations, a canonical variate analysis (CVA) of the symmetric component of shape variation was applied [74]. CVA maximizes the differences between groups relative to the variation within groups and it is therefore one of the most used methods to distinguish among groups [74]. The statistical significance of the pairwise differences in mean shapes was assessed with performing a permutation test of the Procustes distance (10,000 permutations per test).

The amount of symmetric variation was assessed by computing a Procrustes ANOVA as applied in other studies to analyse object symmetry [71], [75], [76]. This test was used to compare the individual-reflection interactions as compared to measurement error, being this latter estimated from the total variation of the entire landmark configuration.

### Climatic Correlation

In order to assess the degree of association between shape and climatic variables, a two block partial least square (PLS) was performed. The geographic coordinates and climatic condition (temperature, rainfall and soil type) (Table 1) were extracted from Croatian Meteorological and Hydrological Service (for complete values since 2009 to 2013 see Table S3), for each one of the analysed sites. PLS was preferred due to the fact that climatic variables are strongly collinear [17]. The two-block PLS analysis is a widely used morphometric method, and it is based on the singular value decomposition of the common covariance matrix of the two variables sets, which in our case consist of the shape and climatic conditions matrices [19], [20]. PLS analysis estimates the correlated pairs of lineal combinations (singular vectors) between these two sets. These singular vectors are generated as pairs of latent variables (one per block), explaining most of the covariance between the original variable blocks, thus maximizing the covariation between the two sets of variables [17], [19], [20]. In order to test the significance of the correlation between the pairs of singular vectors (i.e. climatic variable singular vectors and shape singular vectors), a permutation test with 10,000 rounds randomizations was performed.

### Mantel Test

In order to statistically test whether there was relationship between shape differences and geographic location, a Mantel test was performed. The correlation level was assessed comparing Procrustes (i.e. the morphometric distances between all the wireworms at each location), and geographic (i.e. the geodesic distances between all the locations where the wireworms were sampled) distance matrices by computing their product moment correlation and the Mantel test statistic (observed Z values compared to their permutation distribution after 10,000 rounds).

## Results

**Figure 3:**
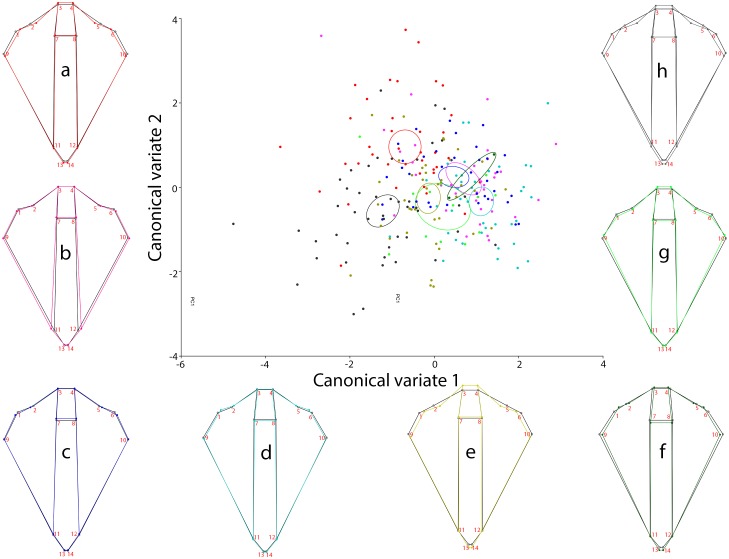
Canonical Variate analysis of the eight *A. ustulatus* populations. The figure shows the firsts two CV axes and the wireframe visualization of the average shape for all populations. a: GAREŠNICA (red) b: LIPIK (pink) c: TOVARNIK (blue) d: BOŠNJACI (light blue) e: SLATINA (dark yellow) f: TEREZINO POLJE (green) g: FERDINANDOVAC (light green) h: OGULIN (black).

**Figure 4:**
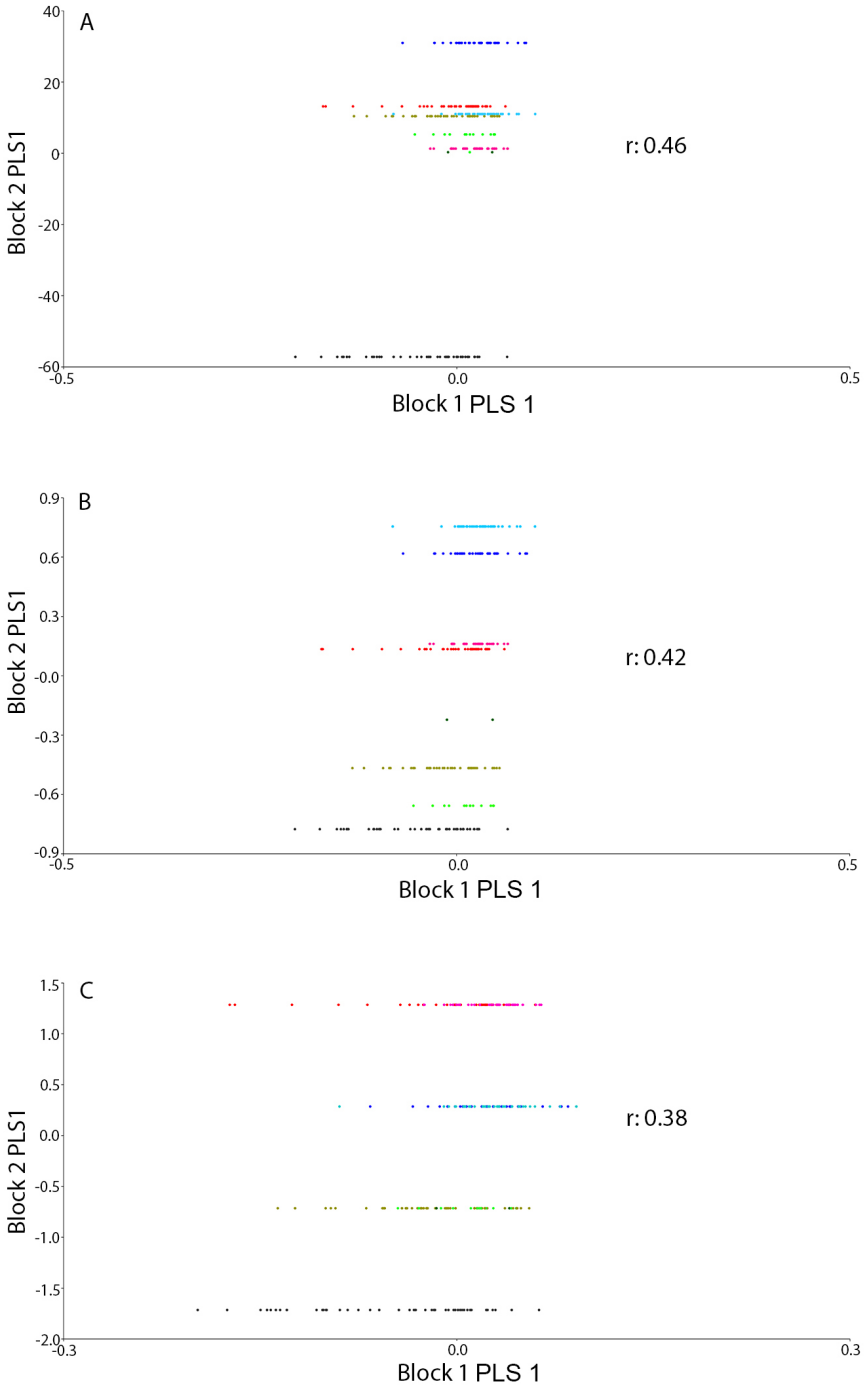
Partial least squares analysis of head shape and environmental condition in *A. ustulatus*. The horizontal axis of the scatter plot is the first PLS axis for head shape and the vertical axis is the first PLS axis for environmental condition for the eight localities. A: temperature, B: Rainfall and C: Soil Type.

**Figure 5:**
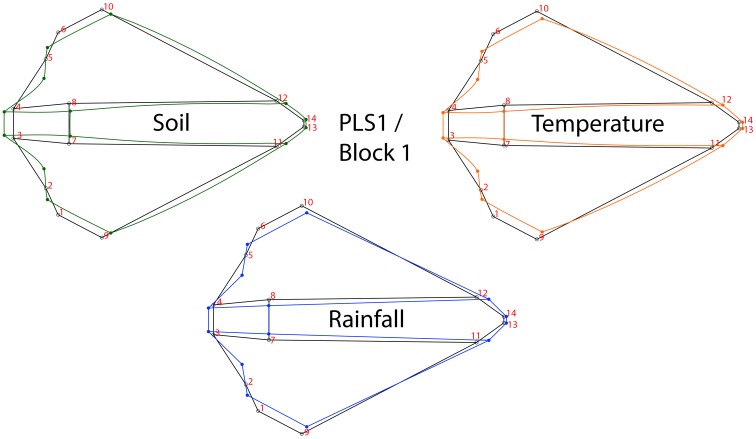
Wireframe visualization of the average shape extracted from the PLS1 associated with the different environmental conditions. The figure colors shows the particular displacements of landmarks relative to each one of the environmental conditions, green: soil, orange: temperature and blue: rainfall.

The Procrustes ANOVA applied to assess the measurement error showed that the mean square for individual variation exceeded the measurement error; therefore it was negligible (Table 2). The same analysis applied to assess the population differences showed significant differences among localities for both size and shape (P<0.0001) (Table 3). The PCA of the residuals of the regression (used to excluded the allometric effects) of the head shape variation showed that the first three PCs accounted for 79.2% (PC1 = 52.8%; PC2 = 17.04%; PC3 = 9.3%) of the total shape variation, hence providing a reasonable approximation of the total amount of variation. The CVA showed a significant differentiation among localities based on Procrustes distances (Fig. 3). The Mantel test indicated that in spite of a relatively high (0.727) correlation value between the morphometric (i.e. Procrustes) and geographic distances (i.e. km), it was not significant (p-value = 0.076; 10,000 permutation rounds). The two-block, partial least-squares analyses of the head shape indicated a moderate association between the shape variables and the environmental ones (Rainfall: r = 0.422, p-value<0.001; Temperature: r = 0.46, p-value<0.001; Type of Soil: r: 0.38, p-value<0.001) (Fig. 4). A strong and significant amount of covariation between the head shape and the environmental variables was graphically visualised by the first block and PLS1 (Fig. 5).

**Table 2:** Measurement error Procrustes ANOVA for both centroid size and shape of *A. ustulatus*, characterised by matching symmetry.

Centroid size					
Effect	SS	MS	df	F	p-value
Individual	4.682	0.142	33	12.1	<0.0001
**Error 1**	**0.374**	**0.012**	**32**		
**Shape**					
Effect					
Individual	0.156	3.95E-04	396	3.09	<0.0001
Side	0.0049	0.0004	12	3.23	0.0002
Ind×Side	0.051	0.0001	396	2.47	<0.0001
**Error 1**	**0.039**	**5.17E-05**	**768**		

Sums of squares (SS) and mean squares (MS) are in units of Procrustes distances (ie. dimensionless).

**Table 3:** Procrustes ANOVA for both centroid size and shape of *A. ustulatus*, characterized by object symmetry.

Centroid size					
Effect	SS	MS	df	F	p-value
Location	7.09	1.013	7	17.31	<0.0001
Individual	14.28	0.058	244		
**Shape**					
Effect					
Location	0.233	0.0028	84	7.17	<0.0001
Individual	1.136	0.0004	2928	3.78	<0.0001
Side	0.009	0.0008	12	8.1	<0.0001
Ind×Side	0.309	0.0001	3012		

Sums of squares (SS) and mean squares (MS) are in units of Procrustes distances (dimensionless) SH: Shape, CS: centroid size.

## Discussion

The eight populations were distinguished according to the relative displacement of the symmetric landmarks 1, 2–5, 6 and 9–10. *A. ustulatus* presents a relatively long larval stage as compared to most insects (ca. 3–5 years) [27]. This interesting life history trait has led to several studies focusing on the effect of soil type on population density or invasive capacity [26], [36], [37], [77]. Nonetheless, there have been no studies trying to assess the influence of soil type (and other climatic conditions) on the cephalic capsule shape. It was expected to contribute to the understanding of the influence of environmental factors on the morphological development of wireworms.

The first dimensions of the PCA accounted for most of the morphological variation, showing that the wireworm head shape has a relative regional ordination (wireframe Fig. 3). This means that each population has relatively different head shape with respect to the others, in such a manner that the majority of the observed shape differences are due to regional differences. The CVA results confirm this interpretation, although there was some overlap between the confidence interval ellipses (Fig. 3). The Mantel test was performed in order to assess whether this regional ordination was expressed in a morphoclinal way (i.e. in accordance with a gradual differentiation relative to distance). Our results did not support the latter perspective, but on the contrary showed that there was no lineal relationship between the shape of the wireworms and the distance between the collection sites. Hence, it was possible to discard the existence of a morphoclinal variation in the larvae head shape. Although, this conclusions must be cautiously considered, since it has been shown that the Mantel test is not always the best method to test for linear relationships [78].

The PLS results showed that the shape variation of the head observed in *A. ustulatus* covaries with the environmental factors. Our study has confirmed a high covariation between the wireworm head shape and the analyzed climatic conditions, although the correlation values between PLS axes were moderate. It is important to keep in mind that these correlations are not the decisive factor that has been maximized by the PLS analysis. In the present study, the shape changes associated to the climatic variables were clearly visible in PLS1, showing that the head shape had an expansion of landmarks 3 and 4 (base of the hair on the mentum) due to soil type differences resulting in a morphotype defined by an elongated head (Fig. 5). The average effect of temperature showed a head shape expansion of landmarks on the maxillary palpi (1–6 and 2–5) and a contraction of the mentum, showing a narrowed head that increasingly expands towards the cardo and alocardo (Fig. 5). Finally the rainfall effect was characterized by a contraction of the landmarks of the maxillary palpi and a moderate elongation of the juncture between the submentum and the cardo (Fig. 5). Nevertheless the shape changes associated with the PLS1 axes showed fairly complex local shifts of landmarks (Fig. 5). Both temperature and rainfall showed similar covariation patterns with respect to shape, exhibiting relative displacements of individual head traits rather than large-scale deformations. However, because the landmark configuration does not include all the traits of the head, this finding should be interpreted cautiously.

It is important to always take into account the possible functional consequences of the observed shape differences when an association between morphology and environment is found [20]. Therefore, the observed shape–environment association noticeable in the first pair of PLS vectors could be interpreted in functional terms. The observed covariation between climatic conditions and shape changes could be a result of the high plasticity of this species. Actually, those populations inhabiting ground water gley soil (i.e. soils were drainage is poor due to a high phreatic surface), have more narrow cephalic capsules (Fig. 3b), suggesting a better penetrating capacity. On the other hand, those individuals occupying wetter muds have more expanded heads (Fig. 3h), allowing them to possibly use it as a shovel in order to dig these kinds of soils in an enhanced way. This plasticity may also explain the capacity of this species as invasive pest, occupying a broad range of host plants both as larva and adult [26], [36]. Previous studies in a diverse range of organisms have shown that phenotypic plasticity can explain the observed morphological differences, especially when considering the ecological distribution of the analysed species [79], [80]. A similar study carried out on the Chrysomelid pest beetle (*Diabrotica virgifera virgifera*), have also established the existence of a relationship between an anatomical structure and soil type [81]. This species exhibit an elongated hind wing morphotype in environments dominated by water gley soil type. By contrast, where a chernozemic or alluvial soil type dominated, a narrow hind wing morphotype was found. Even though the precise mechanism underlying the relationship between *A. ustulatus* and *D. virgifera* phenotypes and soil type is probably different, it is highly suggestive that both are pest species with wide ecological ranges. Perhaps this observed phenotypic plasticity is related to the invasive capacity of this kind of species, although more analyses are needed.

Further geostatistical analyses of spatial patterning and clustering could adjust sampling methods, in order to properly understand the key factors determining population size and pest dynamics [82]. Toepfer et al. [83] have studied the clustering of this group, concluding that this species are environmentally mediated by mortality factors that affect pest populations, which might vary spatially as well as seasonally and due to biological factors such as limited movement of larvae [83]. Hence, it is highly relevant to continue studying the relationship between shape and climatic conditions, but including as well some specific kinematic measurements of the different larvae population displacing through dissimilar soil types. Additionally, future studies should assess the relationship between vegetational structure and climatic conditions (i.e. host plant differences due to climate) and their effect on the morphology of *A. ustulatus* and how this influences its pest dynamics.

## Supporting Information

Table S1 Anatomical Description of the 14th Landmarks in *Agriotes* Schall.
(XLSX)

Table S2 Raw Landmark dataset.
(TXT)

Table S3 Average monthly temperatures and Rainfall.
(XLSX)
