# Correction: Ecomorphological Variation of the Wireworm Cephalic Capsule: Studying the Interaction of Environment and Geometric Shape

**DOI:** 10.1371/journal.pone.0110197

**Published:** 2014-09-30

**Authors:** The PLOS ONE Staff

One of the funders was omitted for this study. Please refer to the complete funding statement below:

This research was supported by the Ministry of Science, Education and Sports of the Republic of Croatia by projects: “The spatial distribution of economically important pests with the use of GIS” (178-1782066-2065) and 09/23 Technology transfer in sugar beet production: improvements in pest control following the principles of integrated pest management (IPM), Croatian Science Foundation. The funders had no role in study design, data collection and analysis, decision to publish, or preparation of the manuscript.

Additionally, the Acknowledgements section is also incomplete. Please refer to the complete Acknowledgements section here:

We thank the two anonymous reviewers that provided us with valuable comments that clearly improved our manuscript. We give great gratitude to Ivana Maruši and Marina Buketa for the technical assistance. We give great gratitude to the Ministry of Science, Education and Sports of the Republic of Croatia by projects: “The spatial distribution of economically important pests with the use of GIS” (178-1782066-2065) and "09/23 Technology transfer in sugar beet production: improvements in pest control following the principles of integrated pest management (IPM), Croatian Science Foundation". HB and TP are grateful with the Becas Chile scholarship program, CONICYT Chile.
